# Dasatinib enhances anti-leukemia efficacy of chimeric antigen receptor T cells by inhibiting cell differentiation and exhaustion

**DOI:** 10.1186/s13045-021-01117-y

**Published:** 2021-07-21

**Authors:** Hao Zhang, Yongxian Hu, Mi Shao, Xinyi Teng, Penglei Jiang, Xiujian Wang, Hui Wang, Jiazhen Cui, Jian Yu, Zuyu Liang, Lijuan Ding, Yingli Han, Jieping Wei, Yulin Xu, Xiaoqing Li, Wei Shan, Jimin Shi, Yi Luo, Pengxu Qian, He Huang

**Affiliations:** 1 Bone Marrow Transplantation Center, The First Affiliated Hospital, School of Medicine, Zhejiang University, No.79 Qingchun Road, Hangzhou, China; 2 Department of Hematology, The Third Affiliated Hospital of Wenzhou Medical University, Wenzhou, China; 3 Center of Stem Cell and Regenerative Medicine, and Bone Marrow Transplantation Center of the First Affiliated Hospital, School of Medicine, Zhejiang University, No. 866 Yuhangtang Road, Hangzhou, China; 4 Institute of Hematology, Zhejiang University and Zhejiang Engineering Laboratory for Stem Cell and Immunotherapy, Hangzhou, China

**Keywords:** Chimeric antigen receptor T cells, Acute lymphoblastic leukemia, Tyrosine kinase inhibitor, Dasatinib, Differentiation, Exhaustion

## Abstract

Relapses of CD19-expressing leukemia in patients who achieved initial remission after CART cell treatment have been reported to correlate with poor CART cells persistence. Sustained tonic signaling or strong activation drives CART cell differentiation and exhaustion, which limit the therapeutic efficacy and persistence of CART cells. Here, we identified dasatinib as the optimal candidate to prevent or reverse both CD28/CART and 4-1BB/CART cell differentiation and exhaustion during ex vivo expansion, which profoundly enhanced the therapeutic efficacy and in vivo persistence. Moreover, strong activation-induced CART cells differentiation, exhaustion and apoptosis driven by CD3/CD28 stimulation or antigen exposure were dramatically prevented or reversed by dasatinib treatment. Mechanistically, dasatinib markedly reduced the phosphorylation of Src and Lck, and downregulated the expression of genes involved in CAR signaling pathways, which resulted in the optimization of cell differentiation, exhaustion and apoptosis-related gene expression. Our study proposes a promising pharmacological approach for optimizing CART cells manufacture, and provides an experimental basis for reinvigorating CART cells in clinical application.

**Supplementary Information:**

The online version contains supplementary material available at 10.1186/s13045-021-01117-y.

**To the editor,**

Chimeric antigen receptor T cells (CART) emerges as a promising therapeutic approach for adoptive immunotherapy of cancer in recent years. The most impressive responses have been achieved in patients with refractory or relapsed B acute lymphoblastic leukemia (B-ALL) by CART cells targeting CD19 [1–3], which provides a potential curative option for patients who are refractory to standard treatments. However, approximately 30–50% of patients experienced leukemia relapse, the majority within one year after CART cells therapy [4]. Relapses of CD19-expressing leukemia in patients who achieved initial remission after CART cell treatment have been reported to correlate with poor CART cell persistence. Sustained tonic signaling or strong activation in manufacture or clinical therapy drives CART cells terminal differentiation [5], exhaustion [6] and even apoptosis, which limits their anti-tumor efficacy and in vivo persistence. Calibrating the activation potential of CAR signaling by modifying the configuration of CD3ζ immunoreceptor tyrosine-based activation motifs (ITAMs) reduced terminal differentiation and exhaustion of CART cells and thus increased their persistence in vivo [7]. Therefore, modulation of T cell activation signaling may be an important and feasible approach to enhance the efficacy and in vivo persistence of CART cells.

The traditional concepts that tyrosine kinase inhibitors (TKIs) inhibit T cell activation [8, 9] inspired us to explore their effects on CART cells differentiation and exhaustion. We systematically evaluated the effects of clinical commonly used TKIs including imatinib, dasatinib and nilotinib on CART cells, and surprisingly identified dasatinib as the potential candidate to strongly reverse differentiation and exhaustion of CD28/CART cells during ex vivo expansion (Fig. 1a, Additional file 1: Fig. S1a-e) with the optimal effective concentration of 30 nM in consideration of relative less impact on cell proliferation (Fig. 1b–d, Additional file 1: Fig. S1f). As expected, Nalm6 bearing mice with treatment of dasatinib pre-treated CD28/CART cells demonstrated significant lower tumor burden and prolonged survival compared to untreated counterparts (Fig. 1e–h). Despite the less tendency of exhaustion, 4-1BB/CART cells exhibited downstream differentiation with prolonged cell culture (Fig. 1i), which also could be effectively prevented in the presence of dasatinib 30 nM without significant impact on cell expansion (Fig. 1j–n), and showed an improved efficacy in xenograft mice models (Fig. 1o–r).

**Fig. 1:**
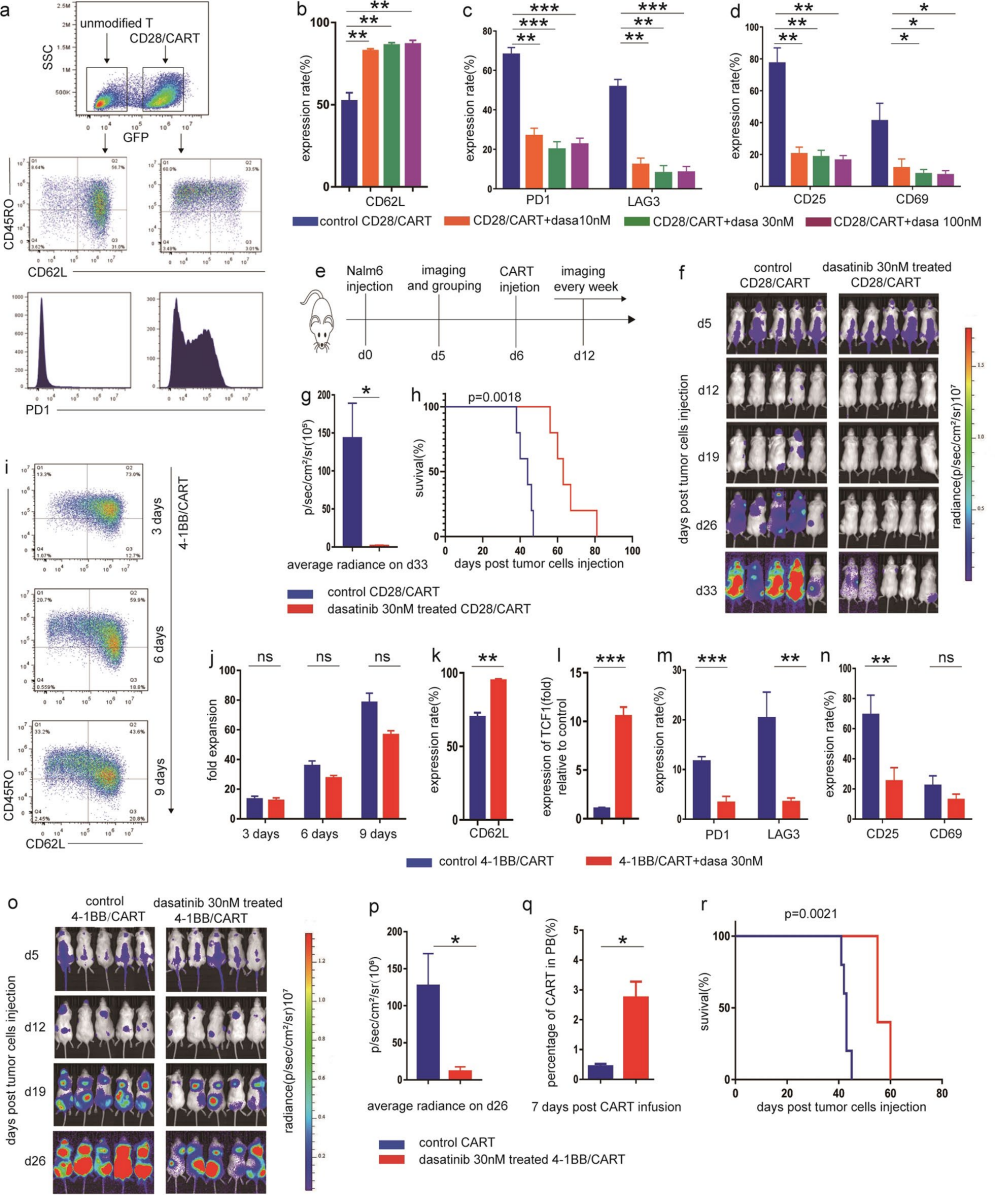
Dasatinib reduced the differentiation and exhaustion of both CD28 and 4-1BB/CART cells during ex vivo expansion, thus enhanced their therapeutic efficacy in mice models. **a** The differentiation and exhaustion in CD28/CART cells were evaluated by expression of CD45RO, CD62L, PD1, TIM3 and LAG3 on 5–7 days after CAR-carrying virus transduction. **b**–**d** 5–7 days after transduction, CD28/CART cells were cultured with dasatinib 10 nM, 30 nM,100 nM and equivalent volume of DMSO to dasatinib in control for 72 h. The quantification of **b** CD62L, **c** PD1, TIM3 and LAG3, **d** CD25 and CD69, respectively, showing the effects of dasatinib on CD28/CART cells differentiation, exhaustion and activation. **e** Schematic diagram of experimental setup depicting that NSG mice were injected with 1 × 10^6^ Nalm6-luc cells followed by administration of 1 × 10^6^ DMSO and 30 nM dasatinib pretreated CD28/CART cells, respectively, 5 days later, and tumor burden was determined by bioluminescent imaging system every week. **f** The dynamics of tumor burden in two groups of Nalm6-bearing mice was assessed by bioluminescent imaging (n = 5 per group). **g** The mean average radiance on representative d33 (n = 5 per group). **h** Dasatinib treated CD28/CART group showed significant prolonged survival compared with control (*p* = 0.0018, log-rank Mantel-Cox test). **i** 4-1BB CART cells were treated with dasatinib 30 nM and equivalent volume of DMSO to dasatinib in control for nine consecutive days, and the expression of CD45RO and CD62L was evaluated on day 3, 6 and 9, respectively. **j** the number of CART cells was calculated by cell counts on day 3, 6 and 9, respectively. **k** The quantification of CD62L on day 9 showing the inhibitory effect of dasatinib on 4-1BB CART cell differentiation with the prolonged ex vivo culture. **l** Real-time PCR was performed to identify differentiation-related transcription factor TCF1. **m** The quantification depicting the expression of inhibitory receptors on day 9. **n** The quantification showing the effect of dasatinib on activation-related markers (CD25 and CD69) in 4-1BB CART cells on day 9. **o** NSG mice were injected with 1 × 10^6^ luciferase expressing Nalm6 on day0, and 5 days later, mice received 1 × 10^6^ DMSO and dasatinib 30 nM pretreated 4-1BB/CART cells, respectively. Tumor burden was determined by bioluminescent imaging every week. **p** The mean average radiance on representative d26 (n = 5 per group). **q** 7 days after CART cells infusion, mice blood was collected, and the percentage of CART cells was determined by flow cytometry. **r** Dasatinib-treated CART group showed prolonged survival compared with control (*p* = 0.0021, log-rank Mantel-Cox test). All data of in vitro experiments was given as n = 3 replicates and representative of three donors; *p < 0.05, **p < 0.01, ***p < 0.001; n.s., not significant; error bars represent the mean ± SEM as determined by a two-tailed unpaired t test

T-cell activation is a necessary step in the manufacture of CART cells, and is also the prerequisite of robust cytotoxicity. However, excessive activation drives CART cells differentiation, exhaustion and even apoptosis. Dasatinib significantly protected CART cells from CD3/CD28 signaling induced apoptosis, differentiation and high expression of inhibitory receptors (eg.PD1 and LAG3) (Fig. 2a–g). Moreover, in differentiated CART cells with high expression of inhibitory receptors induced by tumor antigen exposure (Fig. 2h), dasatinib strongly reversed their stages back to TN and TCM (Fig. 2i), markedly abolished the increased expression of PD1 and LAG3 (Fig. 2j), and promoted cell proliferation (Fig. 2k).

**Fig. 2:**
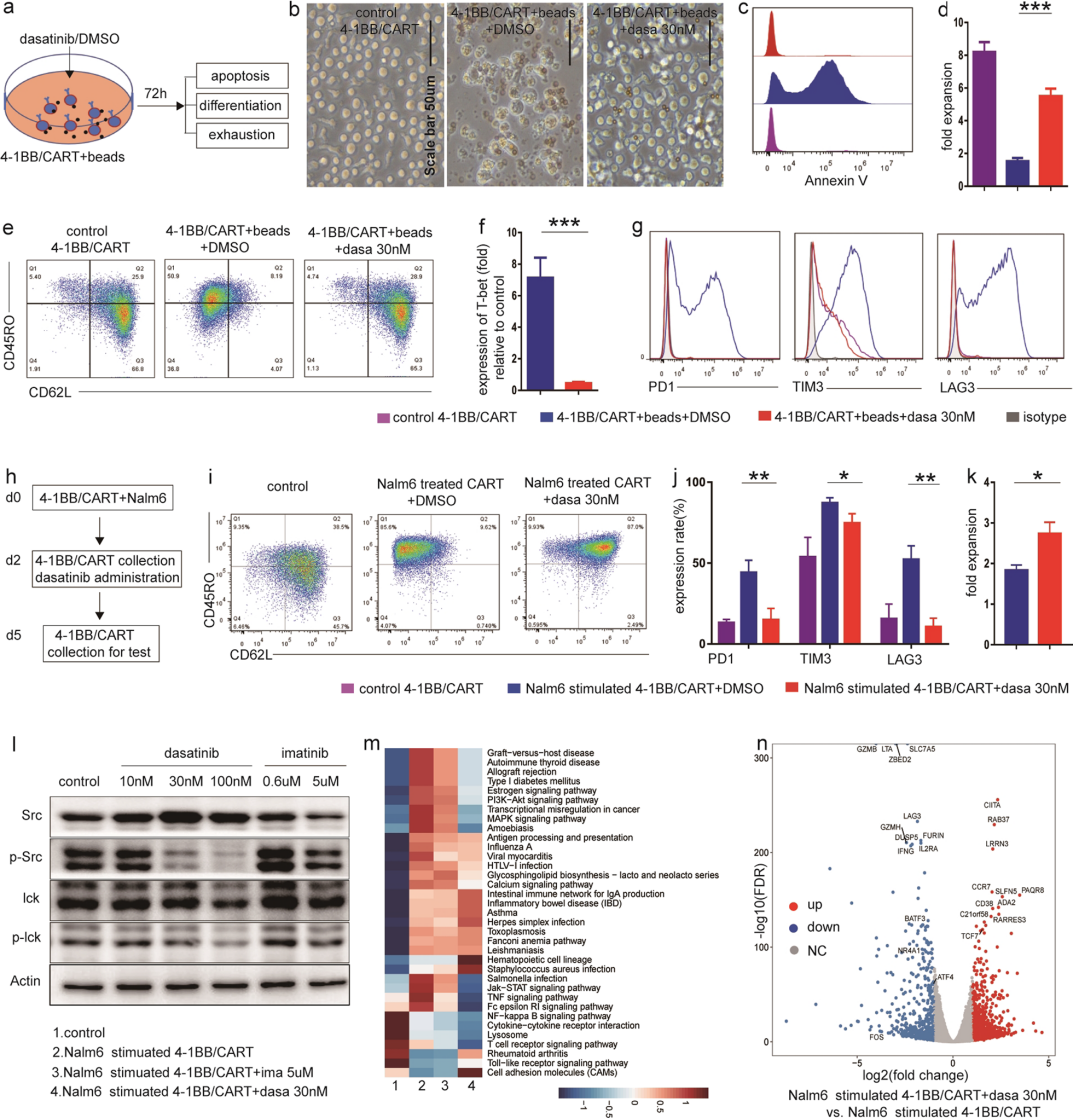
Dasatinib effectively prevented or reversed the strong activation-induced CART cells differentiation and exhaustion by CD3/CD28 stimulation or antigen exposure. **a** Schematic diagram showing CD3/CD28 beads and dasatinib or DMSO were simultaneously added into flow-sorted 4-1BB/CART cells, and the apoptosis, differentiation and exhaustion were evaluated 72 h later. **b** Representative images of CD3/CD28 beads unstimulated, CD3/CD28 beads stimulated and dasatinib-treated CD3/CD28 beads stimulated CART cells. The scale bar represents 50 μm (20 ×). **c** Representative histograms depicting the expression of Annexin V in different groups. **d** Absolute numbers of CART cells were determined and by Cedex XS cell count and calculated for cell expansion. **e** Representative flow cytometry dot plots of CD62L showing the inhibitory effect of dasatinib on 4-1BB CART cell differentiation. **f** Real-time PCR was performed to identify differentiation related transcription factor T-bet. **g** Representative histograms depicting the expression of inhibitory receptors. **h** Schematic diagram showing flow-sorted 4-1BB/CART cells were cocultured with Nalm6 for 48 h, and after eradication of Nalm6, residual CART cells were collected and treated with dasatinib or DMSO for another 72 h. **i** Representative flow cytometry dot plots for CD45RO and CD62L expression in CART cells. **j** Representative histograms depicting the expression of PD1, TIM3 and LAG3 in CART cells. **k** Absolute numbers of CART cells were determined by Cedex XS cell count and calculated for cell expansion in each group. All data mentioned above was given as n = 3 replicates and representative of three donors; *p < 0.05, **p < 0.01, ***p < 0.001; n.s., not significant; mean ± SEM determined by two-tailed unpaired t test. **l** Western blots were performed to determine the protein levels of Src, p-Src(Tyr416), Lck and p-Lck(Tyr505) in common cultured CART cells treated with DMSO (control), different concentration of dasatinib or imatinib (0.6uM for IC50, 5uM for plasma peak concentration [12]) for 15 min. **m** 48 h after coculture of CART cells with Nalm6, residual CART cells were collected and treated with DMSO, dasatinib 30 nM or imatinib 5uM for another 72 h, and then cells were collected for transcriptional profiles. Control represents Nalm6 unstimulated CART cells. GO-term enrichment analysis showing the top list of signaling pathways in each group. **n** Volcano plots showing expression of exhaustion-related regulators (NR4A1, BATF3, ATF4 and FOS), inhibitory receptors (PD1, LAG3), memory-associated transcription factor TCF7 and cell surface marker CCR7 in Nalm6 stimulated CART + dasatinib compared to Nalm6 stimulated CART. Volcano plots were constructed using log2(fold change) and -log10(FDR) values for all genes. Red and blue dots represent genes with more than a twofold change (up or down) in expression and FDR < 0.01

Mechanically, we showed that dasatinib, not imatinib significantly reduced the phosphorylation of Src and Lck (Fig. 2l), downregulated T cell activation associated signaling pathways (T cell receptor, Jak-STAT, MAPK and PI3K-Akt) (Fig. 2m, Additional file 1: Fig. S4b-d), inhibitory receptors (PD1, LAG3) and exhaustion-related regulators (NR4A1, BATF3, ATF4 and FOS), whereas increased expression of naive/memory-associated genes (TCF7, CCR7) (Fig. 2n, Additional file 1: Fig. S3). Besides, p53 signaling pathway which upregulated in Nalm6 stimulated CART cells significantly downregulated in dasatinib treated group (Additional file 1: Fig. S4a). Thus, we reckoned that dasatinib modulates CART cells differentiation, exhaustion and apoptosis by inhibiting cell activation pathway. Interestingly, a recent study demonstrated that transient cessation of CAR signaling by dasatinib could reverse dysfunction and induce epigenetic reprogramming in exhausted CART cells [10]. Future studies are warranted to determine the relationship between epigenetic modifiers, T cell activation, differentiation and exhaustion.

Pharmacologic inhibition of T-cell activation signaling with dasatinib during ex vivo expansion successfully reduced CART cell differentiation and exhaustion, thus enhanced their therapeutic efficacy and in vivo persistence. On the other hand, dasatinib could effectively prevent or reverse the strong activation-induced CART cells differentiation and exhaustion by CD3/CD28 stimulation or antigen exposure, which proposed a potential clinical application of drug for functional reinvigoration of CART cells. Of note, simultaneous application of CART cells with dasatinib limited the efficacy both in vitro and in vivo (Additional file 1: Fig. S2). These findings indicate that the mode and duration of drug administration may be critical for its positive effects on CART cells, which is consistent with the notion proposed by Mestermann et al. [11]. Collectively, dasatinib is a promising pharmacological approach which can be incorporated into CART cells production, and also be potentially applied for functional reinvigoration of CART cells in clinical.

## Supplementary Information

**Additional file 1.** Supplyementary figures and figure legends.

**Additional file 2.** Materials and methods.

## Data Availability

RNA-seq data that support the findings of this study have been deposited in the GEO under accession number GSE151774. All other data supporting the findings of this study are available from the corresponding author upon reasonable request.

## Abbreviations

CAR: Chimeric antigen receptor
scFV: Single-chain variable fragment
B-ALL: B acute lymphoblastic leukemia
CML: Chronic myeloid leukemia
CR: Complete remission
TKI: Tyrosine kinase inhibitor
AICD: Activation-induced cell death
ITAMs: Immunoreceptor tyrosine-based activation motifs
TCR: T cell receptor
PBMC: Peripheral blood mononuclear cells
TN: Naive T cell
TCM: Central memory T cell
TEM: Memory effector T cell
TEF: Effector T cell
DEGs: Differential expressed genes
FDR: False discovery rate
PCA: Principal component analysis
DAVID: Visualization and integrated discovery
GSEA: Gene set enrichment analysis
MAPK: Mitogen-activated protein kinase
JAK/STAT: Janus kinase and signal transducer and activator of transcription
PI3K: Phosphatidylinositol 3 kinase
IL-2: Interleukin-2
PD-1: Programmed death 1
TIM3: T cell immunoglobulin domain and mucin domain 3
LAG3: Lymphocyte activating 3
