# Supplementary material for: Dasatinib enhances anti-leukemia efficacy of chimeric antigen receptor T cells by inhibiting cell differentiation and exhaustion
Source: J Hematol Oncol. 2021 Jul 21;14:113. doi: 10.1186/s13045-021-01117-y (PMC8293573; doi:10.1186/s13045-021-01117-y)
Supplement: Supplementary file 1 — Additional file 1. Supplyementary figures and figure legends. [file 13045_2021_1117_MOESM1_ESM.docx]

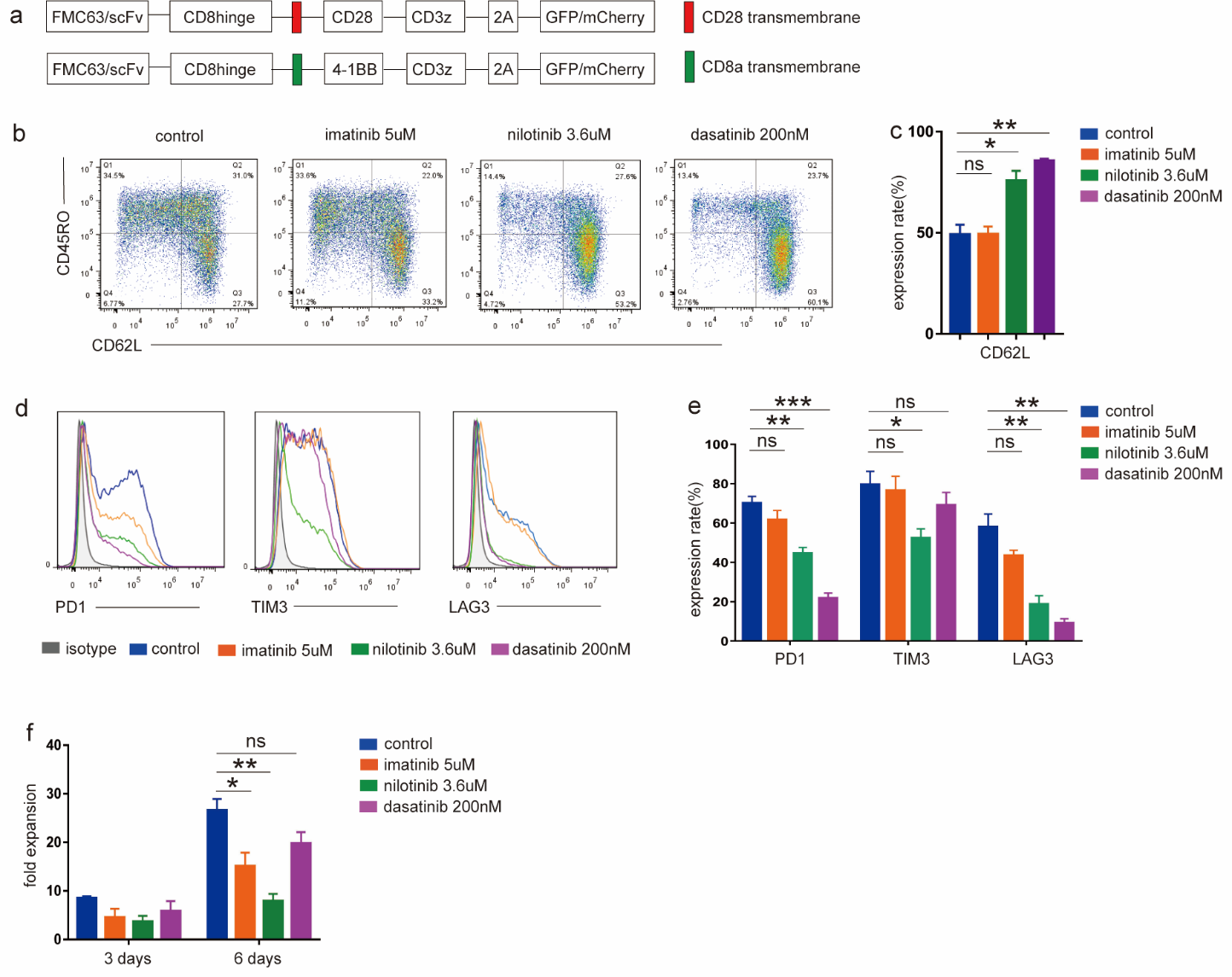


**Supplementary Fig.1.** **Systematic screening of TKIs on therapeutic potency of CD28/CART cells.** (**a**) Schematic representation of CD28 and 4-1BB co-stimulation domain incorporated CAR construction. (**b,c,d,e**) The differentiation and exhaustion in CD28/CART cells were evaluated by expression of CD45RO, CD62L, PD1,TIM3 and LAG3 on 5-7 days after CAR-carrying virus transduction. Then CD28/CART cells were collected and cultured with TKIs including imatinib, dasatinib and nilotinib with the peak plasma concentration of 5uM, 200nM and 3.6uM respectively[1], and equivalent volume of DMSO to TKIs in control for 72 hours. Representative flow cytometry dot plots demonstrating the effects of TKIs on CD28/CART cell differentiation**(b)**, and quantification **(c)** of CD62L expression. Representative histograms (**d**) and quantification (**e**) showing the effects of TKIs on CD28/CART cell exhaustion. Representative of 3 donors. (**f**) The impact of TKIs on CART cell proliferation was determined by cell counting 3 and 6 days after treatment. n=3 replicates; Representative of 3 donors. Error bars represent the mean±SEM as determined by a two-tailed unpaired t test for all data mentioned above. *P <0.05, **P <0.01, ***P <0.001; n.s., not significant.


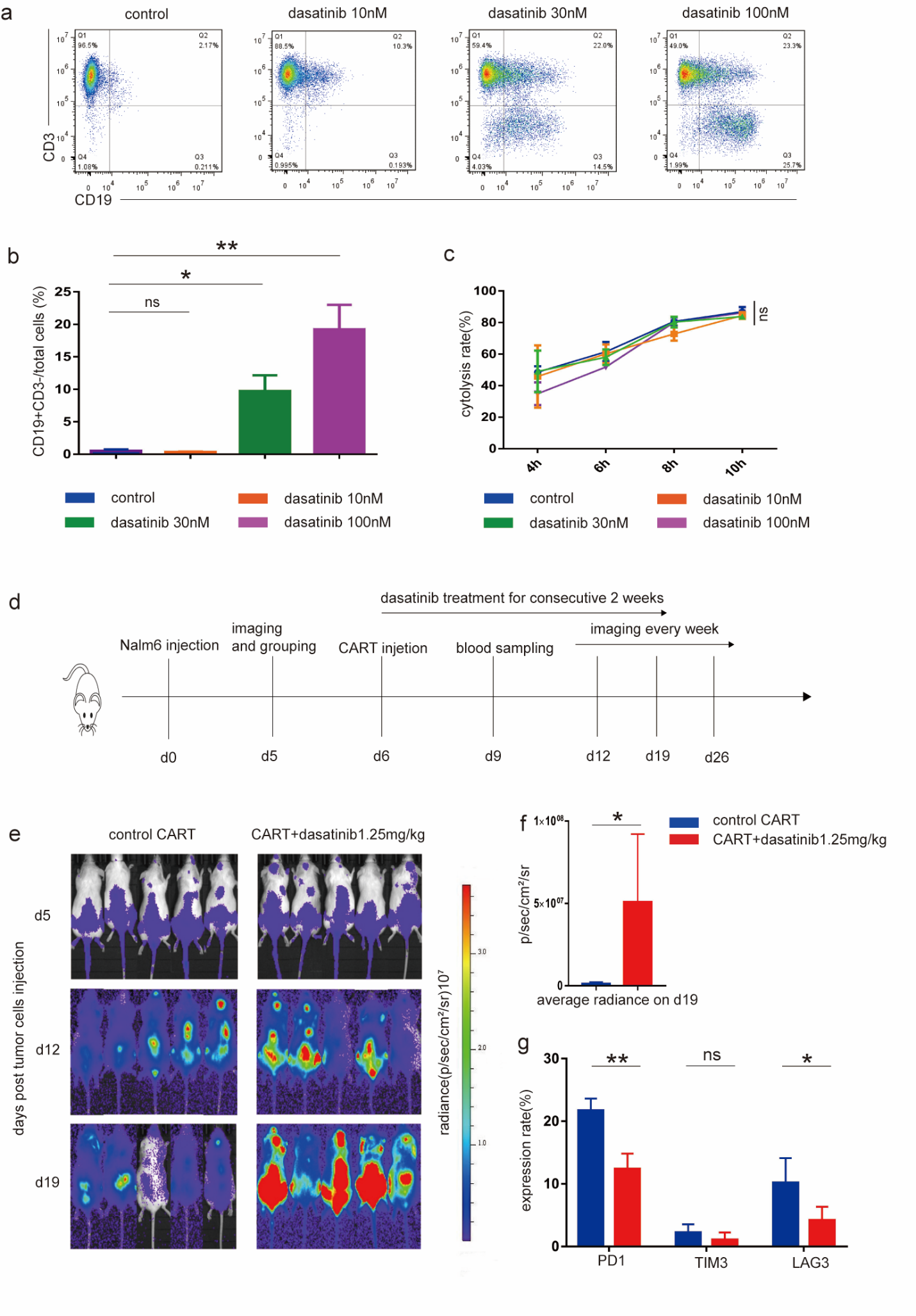


**Supplementary Fig.2. The mode of dasatinib administration determined its effects on CART cells function.** **(a,b)** Coculture of healthy donor derived 4-1BB/CAR T cells and Nalm6 at the ratio of 1:1 in presence of different concentration of dasatinib for 72 hours. Representative flow cytometry dot plots **(a)** and quantification **(b)** showing the function inhibitory effect of dasatinib on CART cells with a dose dependent manner. **(c)** The short-term cytotoxic effect of different concentrations of dasatinib treated CART cells on Nalm6 was determined by luciferase based cytotoxicity assay in the coculture system without the existence of dasatinib. (**d**) Experiment schedule depicts that NSG mice with luciferase expression Nalm6 received 0.5×10^6^ conventional 4-1BB/CART cells at d6, and was simultaneously given dasatinib 1.25mg/kg and vehicle for consecutive 2 weeks respectively for experimental and control group. (**e**) The dynamics of tumor burden in two groups of Nalm6-bearing mice was assessed by bioluminescent imaging (n=5 per group). (**f**) The mean average radiance on representative d19 (n=5 per group). (**g**)The expression of PD1, TIM3 and LAG3 on CART cells in mice peripheral blood 3 days after dasatinib treatment was compared. *P <0.05, **P <0.01, ***P <0.001; n.s., not significant. mean±SEM determined by two-tailed unpaired t test.


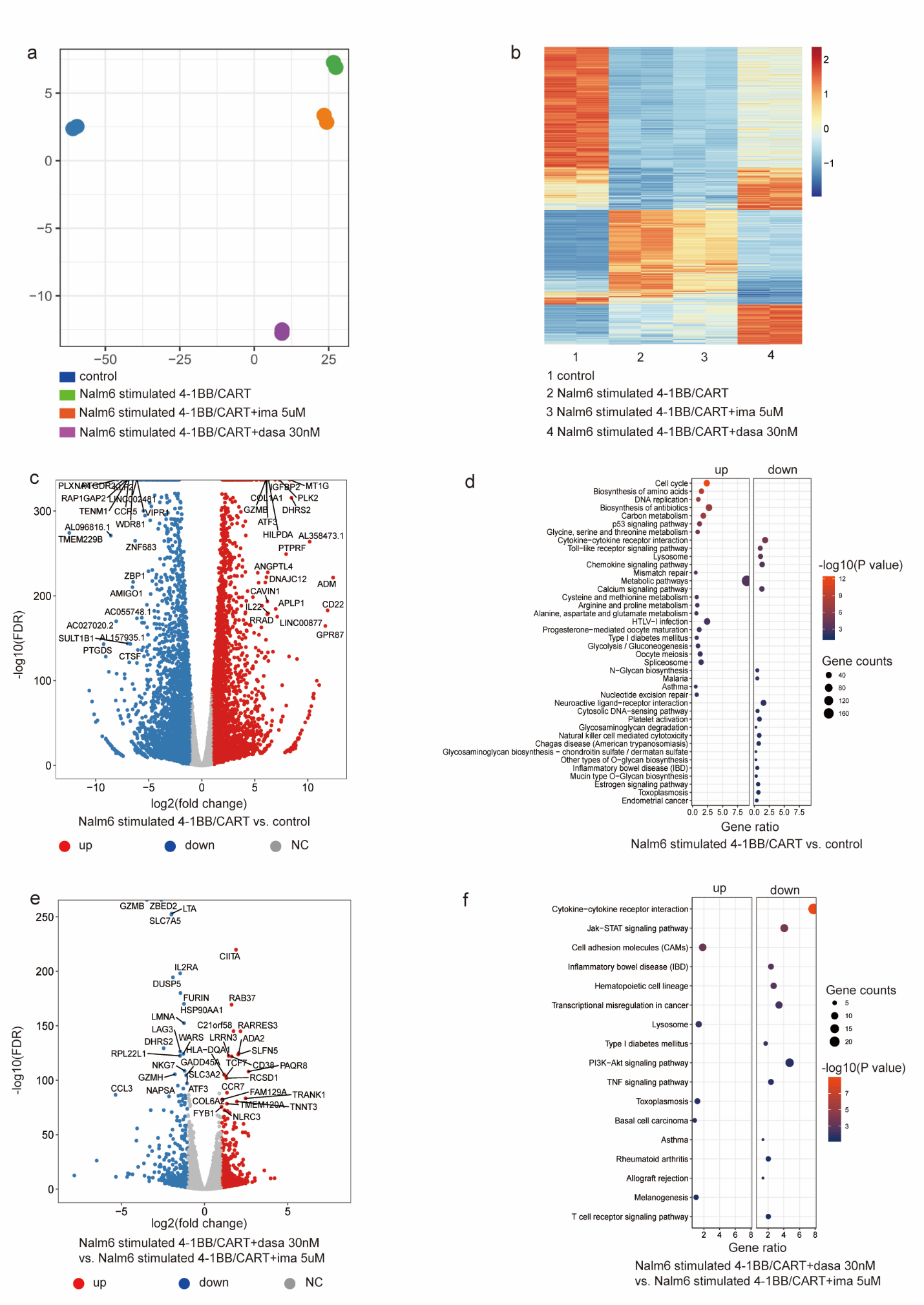


**Supplementary Fig.3. The pairwise comparation of transcriptional profiles.** 48 hours after coculture of 4-1BB/CART cells with Nalm6, residual CART cells were collected and treated with DMSO, dasatinib 30nM or imatinib 5uM for another 72 hours, and then cells were collected for transcriptional profiles. Control represents Nalm6 unstimulated CART cells. (**a**) Principal component analysis (PCA) showing the relationship of transcriptional profiles in control, Nalm6 stimulated 4-1BB/CART, Nalm6 stimulated 4-1BB/CART +dasatinib 30nM and Nalm6 stimulated 4-1BB/CART + imatinib 5uM. (**b**) The heatmap for comparison of the transcriptional profiles of different groups. (**c**) Volcano plots demonstrating the significant change of T cell effector differentiation, activation and exhaustion associated gene expression profiles in Nalm6 stimulated CAR T cells compared to control. (**d**) The signaling pathway in Nalm6 stimulated 4-1BB/CAR T cells and control was compared. (**e**) The comparison of gene expression in imatinib and dasatinib treatment groups. (**f**) The comparison of signaling pathway involving T cell receptor, Jak-STAT, PI3K-Akt was compared in imatinib and dasatinib treatment groups. Volcano plots were constructed using log2(fold change) and–log10(FDR) values for all genes. Red and blue dots represent genes with more than a twofold change (up or down) in expression and FDR < 0.01.


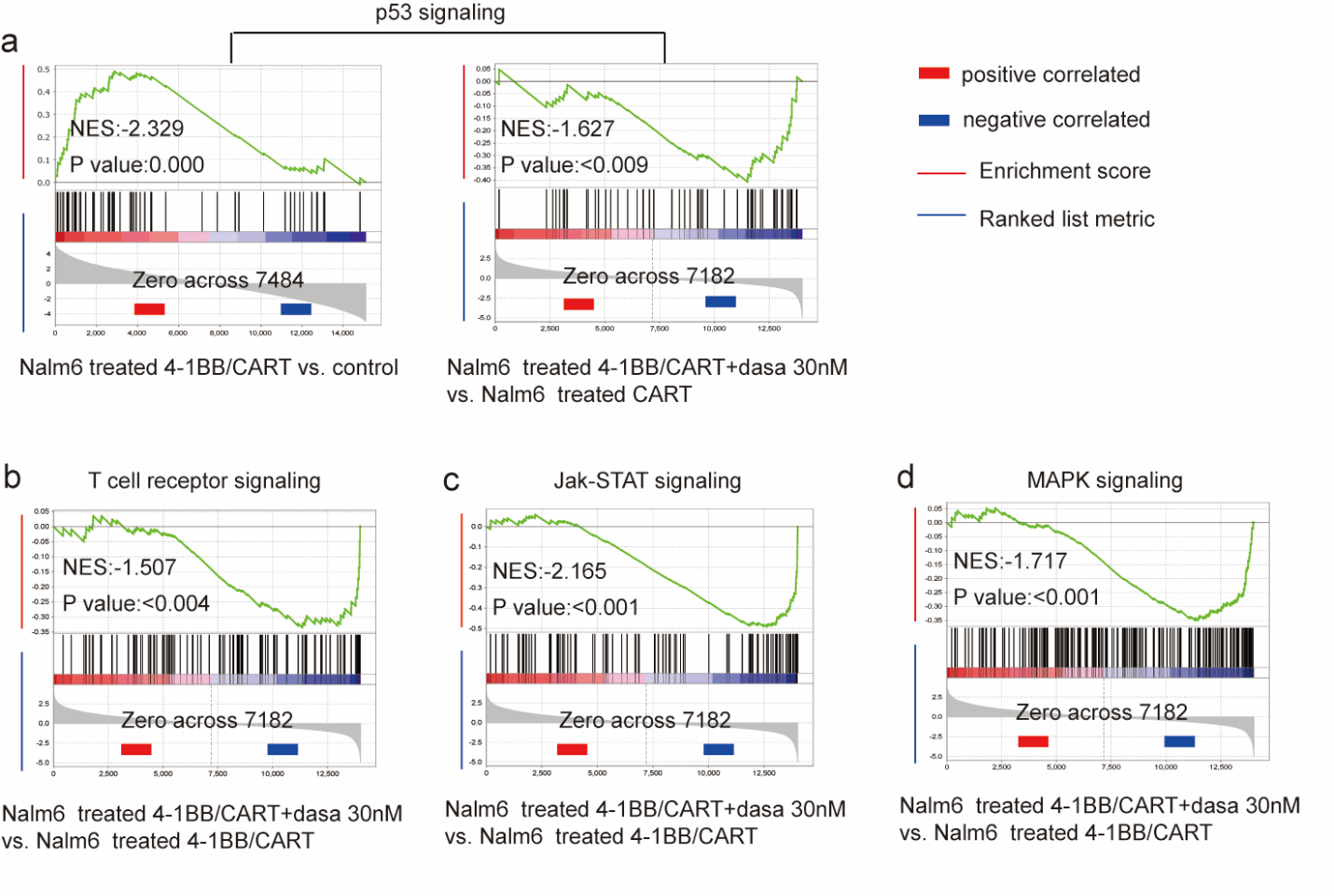


**Supplementary Fig.4. GSEA analysis for the expression profiles of apoptosis and T cell activation associated signaling pathways in dasatinib treated and untreated Nalm6 stimulated 4-1BB/CART cells.** (**a**) GSEA analysis for the expression profiles of p53 signaling pathway. (b,c,d) GSEA analysis for the expression profiles of T cell activation associated signaling pathways (T cell receptor, Jak-STAT, MAPK). The nominal P value and FDR q value were calculated using GSEA software.

**Reference**

1. Wu KN, Wang YJ, He Y, Hu YX, Fu HR, Sheng LX, et al. Dasatinib promotes the potential of proliferation and antitumor responses of human gammadeltaT cells in a long-term induction ex vivo environment. Leukemia. 2014;28(1):206-10.
