# Supplementary material for: Dasatinib enhances anti-leukemia efficacy of chimeric antigen receptor T cells by inhibiting cell differentiation and exhaustion
Source: J Hematol Oncol. 2021 Jul 21;14:113. doi: 10.1186/s13045-021-01117-y (PMC8293573; doi:10.1186/s13045-021-01117-y)
Supplement: Supplementary file 2 — Additional file 2. Materials and methods. [file 13045_2021_1117_MOESM2_ESM.docx]

**Methods**

**Mice**

NSG mice (NOD- PrkdcscidIL2rgtm1/Bcgen) were obtained from Biocytogen. Age and sex matched mice at the age of 5–8 weeks were used in these studies. All mice were housed at the center for drug safety evaluation and research of Zhejiang University under pathogen-free conditions, and all procedures were approved by the ethical committee of Zhejiang University.

**Cell lines**

Nalm6 and Nalm6 transduced with GFP/luciferase (Addgene) were cultured in RPMI-1640 (Corning) and with 10% FBS (Gibco), 100 U/ml penicillin and 100μg/ml streptomycin (Life Technologies).293T cells were cultured in DMEM (Corning) and with 10% FBS (Gibco).

**CAR construct design**

The scFv sequence specific for CD19 was derived from Clone FMC63 as previously described [1]. The anti-CD19 scFv sequence was inserted in tandem with the human CD8/CD28 transmembrane, CD8 hinge, 4-1BB/CD28 costimulatory domain, CD3z intracellular regions and T2A-GFP or mCherry sequence. In this study, CAR T cells incorporating CD28 co-stimulatory domain were used in systematic screening of TKIs on reversing CAR T cells differentiation and exhaustion, the determination of dasatinib’s optimal concentration and the mice associated experiments in Fig.1, and CAR T cells incorporating 4-1BB co-stimulatory domain were used for all other experiments.

**Lentivirus production**

CD28/CAR and 4-1BB/CAR-encoding lentiviral supernatants were produced via transient transduction of the 293T cell line. Briefly, 293T cells were transduced via Polyethylenimine (Polysciences) with the plasmids encoding the CAR and envelope protein psPAX2, pMD2.G. Supernatants were collected 48 and 72 hours after transduction, and prepared into 10^8^ virus particle/ml after ultracentrifugation (Beckman).

**T cell isolation and lentiviral transduction**

Human PBMCs were isolated from peripheral blood of healthy donors at the First Affiliated Hospital of Zhejiang University. Ethical permission was granted by the Clinical Trial Ethics Committee of the First Affiliated Hospital of Zhejiang University. T cells were enriched and activated with anti-CD3/CD28 beads (Life Technologies) in the ratio of cell:bead=1:3 for 24 hours prior to virus transduction. Condensed viral supernatant was used for transduction in accordance with MOI (multiplicity of infection) =10.

**CAR-T cell culture**

Transduced T cells were cultured and expanded in RPMI with 10%FBS, 100 U/ml penicillin ,100μg/ml streptomycin and 100IU/ml IL-2 (Peptech). Three days after transduction, anti-CD3/CD28 beads were removed. Culture medium and IL-2 were changed every 2 days.

**Treatment of tyrosine kinase inhibitors (TKIs)**

For experiments of the impacts of TKIs on CART cells ex vivo culture, CD28/CART cells were cultured with RPMI with 10%FBS and 100IU/ml IL-2 for 5-7 days after transduction and treated with dasatinib (Sigma), nilotinib (Sigma) and imatinib (Sigma) for 3 days, then analyzed. 4-1BB/CART cells were cultured with RPMI with 10%FBS and 100 IU/ml of IL-2 for 3-5 days after activation and treated by dasatinib (Sigma) for consecutive 9 days with replacement of medium and dasatinib every 3 days, then analyzed. For experiments of the impacts of dasatinib on CART cell apoptosis, differentiation and exhaustion induced by anti-CD3/CD28 beads, GFP+ 4-1BB/CART cells were sorted at 3-5 days after transduction and expanded for another 3–7 days, then treated with anti-CD3/CD28 beads by the ratio of beads to cells 1:1 and different concentrations of dasatinib simultaneously for 72 hours. For experiments of the impacts of dasatinib on differentiation and exhaustion of CART cells stimulated by Nalm6, GFP+ 4-1BB/CART cells were sorted at 3-5 days after transduction and expanded for another 3-7 days, then treated with Nalm6 at the ratio of Nalm6 to CART 1:1 for 48 hours, and then dasatinib was added for another 72 hours. All TKIs were dissolved in DMSO (Sigma) and prepared in different concentrations. And equivalent volume of DMSO to TKIs was used in control.

**Flow cytometry**

For ex vivo experiment CART cells were stained with following conjugated antibodies: Annexin V, CD3, CD19, CD25, CD69, PD1, TIM3, LAG3, CD45RO, CD62L and matched isotype controls (Biolegend). CytoFLEX (Beckman) was used with FlowJo 7.6 software for analysis. All FACS plots presenting CART cell phenotype data were conducted on gated GFP or mCherry positive CART cells. Positive/Negative populations were determined by isotype. The subsets of CART cells were defined as TN (CD45RO−/CD62L+), TCM (CD45RO+/CD62L+), TEM (CD45RO+/CD62L−) and TE(CD45RO−/CD62L−). Flow sorting was performed on gated GFP or mCherry positive CART cells with a Beckman moflo Astrios EQ (Beckman).

**Co-culture killing assay**

CART cell cytolytic ability was evaluated by Luciferase based cytotoxicity assay. Target luciferase expressing NALM6 cells were co-incubated with CART cells for 4 hours at effector-to-target (E:T) ratios ranging from 1:1 to10:1. Harvest mixed cells and added with reagent of Bright-GloTM Luciferase Assay system (Promega) for 2 minutes to allow complete cell lysis, and measure the cell viability in a luminometer. Cytotoxicity efficiency was calculated as: lysis% = (T cell viability − CART cell viability)/ (T cell viability) × 100%.

**Western blots**

CART cells were treated with dasatinib or imatinib for 15 min. 3×10^6^ washed cells were lysed in 100 μl of RIPA buffer (PBS, 1% NP40, 0.5% sodium deoxycholate, 0.1% sodium dodecyl sulfate) with 1×Complete EDTA-free protease inhibitor (Roche) and 0.5 mM sodium vanadate (New England BioLabs), and then incubated for 30 min on ice. Western blots were then performed on supernatants of centrifuged samples, using anti-Src antibody and anti-p-Src(Tyr416) antibody (Cell Signaling Technology), anti-Lck and anti-p-Lck(Tyr505) antibody (Cell Signaling Technology) and CleanBlot-IP Detection Reagent (Thermo Scientific).

**Real-time PCR**

mRNA was extracted from control and treated CART cell groups according to the manufacturer’s protocol. And reversing transcribed into cDNA using the Super Script First-Strand Synthesis System (Life Technologies). All reactions were performed with TaqMan Fast Universal PCR Master Mix (Applied Biosystems) on an Applied Biosystems Step One Plus real-time PCR machine, using the following primers,

T-bet (F: GAAACCCAGTTCATTGCCGT, R: CCCCAAGGAATTGACAGTTG).

TCF1(F: CTGGCTTCTACTCCCTGACCT, R: ACCAGAACCTAGCATCAAGGA).

**Murine xenograft models**

Mice were randomly assigned into treatment groups and all experiments were performed with at least 5 mice per group. The mice were injected with 1×10^6^ luciferase/GFP/Nalm6 cells though tail vein, and 1×10^6^ dasatinib treated or untreated mCherry/CART cells were administered 5 days later. Tumor burden was determined by bioluminescent imaging system (IVIS Luminu III, Perkin-Elmer) 24 hours prior to CART cells transfer and performed weekly. Peripheral blood was harvested at 7 days after CART cells administration and analyzed by flow cytometry.

**Sample preparation and data analysis for RNA-sequencing**

Total RNA was extracted from CART cells in control and treatment groups according to the manufacturer’s protocol. cDNA libraries were constructed using the NEBNext® UltraTM RNA Library Prep Kit (Illumina) and sequenced on an Illumina Hiseq platform and 150 bp paired-end reads were generated. Clean RNA-Seq reads for each sample were aligned to the human reference genome GRCh38 (ENSEMBL release 94) by HISAT2 with default setting[2]. FeatureCounts was used to quantify genes expression level with parameter “-Q 10 -B -C”[3]. Differential expressed genes (DEGs) were determined by edger with a fold change >2 and false discovery rate (FDR) < 0.05[4]. Principal component analysis (PCA) was performed by R package FactoMineR. Database for Annotation, Visualization and Integrated Discovery (DAVID) v6.8 web tool (https://david.ncifcrf.gov/) were used to perform GO and KEGG enrichment analyses with a significance of P < 0.05[5]. Gene set enrichment analysis (GSEA) was performed to explore whether identified sets of genes showed significant differences between two groups using the C2-KEGG collection from MSigDB[6].

**Statistical analysis**

All experiments were repeated for 3 times and CAR-T cells generated from 3 individual healthy donors. Figures are representatives of at least 3 individual experiments. Mean ± SEM was used in statistical analysis which was performed with GraphPad Prism 7. For comparisons between two groups, a two-tailed unpaired t-test was used; For comparisons of survival between two groups, log-rank Mantel-Cox test was used.

**References**

1. Zola H, MacArdle PJ, Bradford T, Weedon H, Yasui H, Kurosawa Y. Preparation and characterization of a chimeric CD19 monoclonal antibody. Immunology and cell biology. 1991;69 ( Pt 6):411-22.

2. Kim D, Langmead B, Salzberg SL. HISAT: a fast spliced aligner with low memory requirements. Nature methods. 2015;12(4):357-60.

3. Liao Y, Smyth GK, Shi W. featureCounts: an efficient general purpose program for assigning sequence reads to genomic features. Bioinformatics. 2014;30(7):923-30.

4. Robinson MD, McCarthy DJ, Smyth GK. edgeR: a Bioconductor package for differential expression analysis of digital gene expression data. Bioinformatics. 2010;26(1):139-40.

5. Huang da W, Sherman BT, Lempicki RA. Bioinformatics enrichment tools: paths toward the comprehensive functional analysis of large gene lists. Nucleic Acids Res. 2009;37(1):1-13.

6. Subramanian A, Tamayo P, Mootha VK, Mukherjee S, Ebert BL, Gillette MA, et al. Gene set enrichment analysis: a knowledge-based approach for interpreting genome-wide expression profiles. Proc Natl Acad Sci U S A. 2005;102(43):15545-50.
